# Modified pressure cooker vs. push-and-plug technique in transarterial embolization for brain arteriovenous malformations: a retrospective comparative study

**DOI:** 10.3389/fneur.2025.1643136

**Published:** 2025-10-10

**Authors:** Haojie Wang, Bingsen Xie, Ye Xu, Yibin Zhang, Peisen Yao, YuanXiang Lin, Shufa Zheng, Dezhi Kang

**Affiliations:** ^1^Department of Neurosurgery, Neurosurgical Research Institute, First Affiliated Hospital, Fujian Medical University, Fuzhou, China; ^2^Department of Neurosurgery, National Regional Medical Center, Binhai Campus of the First Affiliated Hospital, Fujian Medical University, Fuzhou, China; ^3^Fujian Provincial Clinical Research Center for Neurological Diseases, The First Affiliated Hospital, Fujian Medical University, Fuzhou, China; ^4^Fujian Provincial Institutes of Brain Disorders and Brain Sciences, The First Affiliated Hospital, Fujian Medical University, Fuzhou, China

**Keywords:** brain arteriovenous malformation, pressure cooker technique, transarterial curative embolization, Spetzler–Martin grade, push-and-plug technique

## Abstract

**Objective:**

This study retrospectively analyzed patients with brain arteriovenous malformation (bAVM) treated by transarterial curative embolization using either the modified pressure cooker technique (mPCT) or the conventional push-and-plug technique (PPT). The primary objective was to assess mPCT’s safety and efficacy by comparing occlusion rates and complications with PPT.

**Materials and methods:**

Data were retrospectively collected from all bAVM patients who underwent transarterial curative embolization at our institution between April 2019 and April 2023. A total of 188 patients were included, with 61 treated using the mPCT and 127 with the PPT. Baseline characteristics, angiographic and clinical outcome, complications and follow-up data were systematically evaluated and analyzed. Lesions were categorized into two groups according to Spetzler–Martin grade (SMG): SMG I-III and SMG IV-V. Furthermore, multivariable logistic regression was performed to identify independent risk factors for incomplete obliteration.

**Results:**

Baseline characteristics were well balanced between the two groups. Immediately after the procedure, the mPCT group achieved a complete obliteration rate of 49.2% compared with 34.6% in the PPT group (*p* = 0.056). Subgroup analysis demonstrated a markedly higher immediate complete obliteration rate in SMG I-III lesions treated with mPCT (62.5% vs. 40.1%; *p* = 0.018). Overall complication rates did not differ significantly between groups (19.7% vs. 23.6%; *p* = 0.543). On multivariable analysis restricted to the mPCT group, nidus size >3 cm was an independent predictor of incomplete obliteration (OR = 14.042, 95% CI: 2.126–92.739; *p* = 0.006).

**Conclusion:**

Overall, occlusion rates did not differ significantly between mPCT and PPT, but mPCT achieved higher rates in SMG I–III lesions with comparable complication rates, suggesting potential benefits in selected cases that require confirmation in larger prospective cohorts with longer follow-up.

## Introduction

Brain arteriovenous malformations (bAVMs) represent abnormal direct shunts between cerebral arteries and veins, converging into a vascular nidus prone to rupture, which can result in life-threatening intracranial hemorrhage and long-term disability (1). The incidence of bAVMs is estimated at 1.10–1.42 cases per 100,000 individuals, with a higher prevalence among adolescents and young adults aged 20–40 (2). The overarching therapeutic goal is the complete obliteration to prevent future hemorrhages. Current treatment modalities include microsurgery, radiosurgery, embolization, or a combination of these therapies (3–5).

Endovascular embolization has advanced considerably over the past two decades, particularly with the adoption of the liquid embolic agent ethylene vinyl alcohol copolymer (Onyx, Medtronic, MN, United States) (6). Onyx’s non-adhesive properties and extended polymerization time enable controlled, slower filling and deeper nidus penetration, thereby enhancing the potential for curative embolization in selected bAVM cases (7, 8). Katsaridis et al. (9) reported cure rates approaching 53.9% using Onyx alone, while Saatci et al. (10) achieved complete occlusion in 51% of 350 patients. Nonetheless, Onyx presents technical challenges for operators, including the risk of microcatheter entrapment or obstruction, with reported incidence ranging from 0.0 to 9.7% and an average rate of 5.8% (11).

Traditionally, Onyx is delivered by slowly creating a proximal plug to minimize reflux, a strategy termed the push-and-plug technique (PPT) (12). However, repeated cycles of injection with waiting may interrupt forward progression of Onyx through the nidus, sometimes leading to premature termination of the procedure (13). Insufficient reflux control can also result in unintended arterial occlusion or catheter obstruction, thereby increasing the risk of ischemic complications (14).

To address these challenges, Chapot et al. (15) and Ierardi et al. (16) introduced the Pressure Cooker Technique (PCT), which creates a proximal plug to achieve stable reflux control and allows continuous Onyx injection into the nidus. Since its introduction, several modifications of the original PCT have been described with encouraging results. The classical PCT involves detachable coils followed by injection of n-butyl cyanoacrylate (NBCA) to establish a permanent proximal plug, whereas the simplified PCT (sPCT) relies solely on nylon stretch-resistant fibred helical coils or extra-soft Kaneka coils (17, 18) and other modified PCT (mPCT) variants may use NBCA alone or reverse the sequence of plug formation (16, 19). The present study describes another modification of PCT, in which detachable platinum coils are first deployed and subsequently reinforced with a small amount of Onyx to secure the plug, thereby improving placement flexibility, stability, and sealing integrity.

Reports on proximal plugs created with coils and Onyx remain limited, and the available case numbers are small. Further clarification of their potential advantages and drawbacks is warranted (20). Against this background, this study retrospectively analyzed patients treated at our center with transarterial curative embolization using either the mPCT or the PPT. The primary objective of this study was to directly compare the complete occlusion rates achieved with mPCT versus PPT, both at immediate post-procedural angiography and at short-term follow-up, and to further evaluate their safety, efficacy, and predictors of angiographic outcomes.

## Materials and methods

### Patients

**Figure 1:**
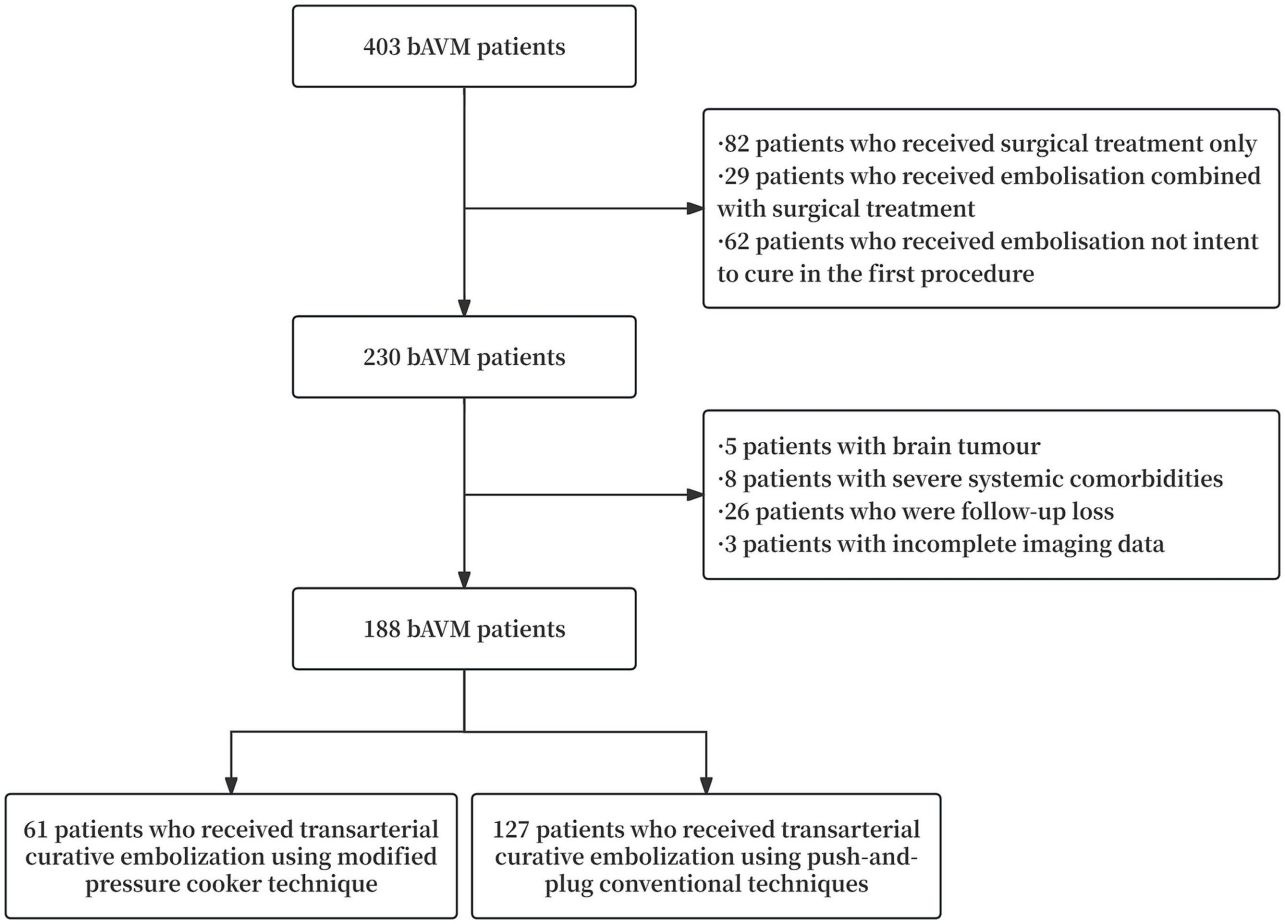
Flowchart of patient inclusion.

This retrospective study collected clinical data from patients with bAVM treated at the First Affiliated Hospital of Fujian Medical University, in accordance with to the Declaration of Helsinki and approval from the institutional Ethics Committee. A total of 188 patients treated between April 2019 and April 2023 were included based on the following criteria: (1) bAVM confirmed by magnetic resonance angiography (MRA), computed tomography angiography (CTA), or digital subtraction angiography (DSA); (2) treatment exclusively by endovascular embolization; and (3) use of Onyx 18 with the intent to achieve complete nidus occlusion during the initial procedure. Exclusion criteria were: (1) treatment with non-curative embolization or combined therapies such as surgery or radiotherapy; (2) coexisting cavernous malformations or moyamoya disease; (3) concurrent cranial tumors; (4) history of systemic diseases; and (5) incomplete clinical data. A flowchart of the study is presented in Figure 1.

### Clinical data

Demographic and medical data, including sex, age, clinical presentation, medical history, and modified Rankin Scale (mRS) scores at admission, were systematically collected. Angioarchitectural features of bAVMs were evaluated, including nidus size, diffuseness, location, venous drainage pattern, presence of perforating arterial feeders, associated aneurysms, and concomitant pial or dural AVMs. Lesions were classified using the Spetzler–Martin grading (SMG) system, with grades I–III defined as “low grade” and grades IV–V as “high grade,” following Julien Burel’s classification (21). Patients were allocated to two groups according to treatment strategy, and outcomes after the initial procedure were compared between groups. All evaluations were conducted by an experienced team of interventional neuroradiologists and clinical staff.

### Procedure of mPCT technique

**Figure 2:**
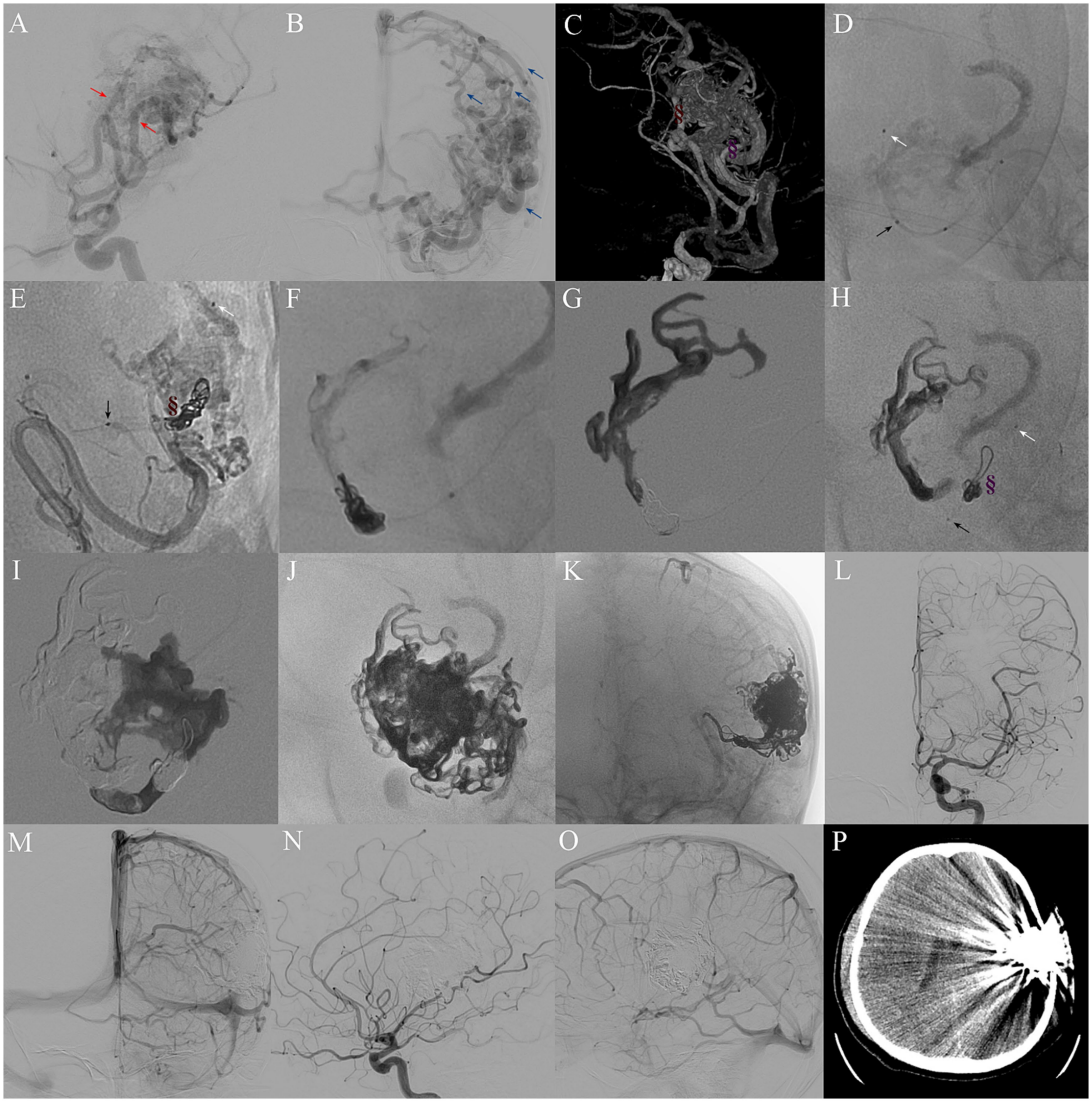
Procedure of mPCT embolization for complete nidus occlusion. **(A)** Preoperative DSA indicating a 5.2 cm bAVM nidus, supplied by two main feeders from the left middle cerebral artery (red arrows) and one feeder from the left anterior cerebral artery, and **(B)** drained by cortical venous to the superior sagittal sinus and also drained downwards into the basilic vein (blue arrows). **(C)** Location of two plugs comprising coils and Onyx in feeders (brown and purple symbols). **(D,E,H)** Single shot fluoroscopy showing the markers of two microcatheter tips: Apollo (white arrow) and Wissky (black arrow) in the same selective feeder. **(E–G)** Formation of a coil-Onyx plug (brown symbol) to control reflux. **(H,I)** Selection of another feeder for a second mPCT embolization. **(J,K)** Post-embolization DSA revealing Onyx cast. **(L–O)** Immediate postoperative selective left ICA DSA confirming complete nidus exclusion. **(P)** Postoperative brain CT showing no hemorrhage or infarction.

All procedures were performed under general anesthesia using a biplane flat-panel angiographic suite (Artis Q; Siemens). After administering 3,000 IU of heparin intravenously, selective intracranial vessel catheterization was performed. A 6F guiding catheter was advanced via femoral arterial access to the target artery, and selective DSA was performed. As shown in Figure 2, the mPCT involves placement of a detachable microcatheter (Apollo, Medtronic; Sonic, Balt) close to the nidus and positioning a non-detachable microcatheter (Wissky, Ricoton; 1.7F) between the distal marker and the detachable point. Through the non-detachable catheter, platinum microcoils were deployed to create the proximal plug, followed by the injection of a small volume of Onyx, ensuring only minimal permeation into the coils without entering the nidus. The number of coils was adjusted according to the diameter of the feeder vessel. Onyx was then injected slowly under fluoroscopic guidance, with pauses if reflux occurred allow formation of form a stable barrier. The volume of Onyx required was dependent on the number of coils deployed to achieve a stable plug. Some cases required multiple embolization cycles (22). After completion of embolization and microcatheter removal, global angiography and Dyna CT were performed. In selected patients, adjunctive PPT embolization was additionally employed to achieve complete nidus occlusion.

### Outcome assessment

Patients were managed according to established bAVM guidelines (23). Clinical outcomes were assessed up until death or 6 months after discharge using the mRS. A good outcome was defined as an mRS score of 0–2, whereas a poor outcome was defined as 3–6. Angiographic follow-up at 3 months was performed with DSA, and complete radiological obliteration was defined as the absence of the nidus and early draining veins on arterial, capillary, and venous phases.

### Statistical analysis

Statistical analyses were performed using SPSS (v27.0, IBM Corp.) and GraphPad Prism (v8.3.0). Continuous variables were expressed as mean ± standard deviation (SD), and categorical variables as frequency and percentages. The Student’s *t*-test and chi-square or Fisher’s exact test were used for comparisons. Receiver operating characteristic (ROC) curve analysis was applied to identify predictive indicators and determine optimal cutoff values. Variables with *p* < 0.10 in univariable analysis were entered into multivariable logistic regression to identify factors associated with incomplete obliteration. Odds ratios (ORs) with corresponding 95% confidence intervals (CIs) were reported, and statistical significance was defined as *p* < 0.05.

## Results

### Patient and bAVM characteristics

A total of 188 patients were included, with 113 males (60.1%) and 75 females (39.9%), and a mean age of 34.92 ± 16.23 years. As summarized in Table 1, there were no marked differences between the groups in age, gender, clinical presentation, medical history, SMG, or mRS scores at admission (all *p* > 0.05). Likewise, nidus characteristics, including size, location, eloquence, compactness, morphology, and vascular features, were also comparable between groups (all *p* > 0.05).

**Table 1:** Basic clinical and angioarchitectural characteristics of patients treated with mPCT versus PPT.

Variable	Totals *n* = 188	mPCT-group *n* = 61	PPT-group *n* = 127	*p*-value
Sex				0.397
Male	113(60.1%)	34(55.7%)	79(62.2%)	
Females	75(39.9%)	27(44.3%)	48(37.8%)	
Age, yrs	34.923(16.227)	34.393(15.793)	35.177(16.487)	0.757
Clinical presentation				0.590
Headache	54(28.7%)	20(32.8%)	34(26.8%)	
Seizures	36(19.1%)	11(18.0%)	25(19.7%)	
Hemorrhage	77(41.0%)	26(42.6%)	51(40.2%)	
Neurological deficit	9(4.8%)	1(1.6%)	8(6.3%)	
Incidental	12(6.4%)	3(4.9%)	9(7.1%)	
Medical history				
Smoking	41(21.8%)	14(23.0%)	27(21.3%)	0.793
Drinking	26(13.8%)	10(16.4%)	16(12.6%)	0.480
Hypertensive	27(14.4%)	11(18.0%)	16(12.6%)	0.320
Diabetes	22(11.7%)	7(11.5%)	15(11.8%)	0.947
Hemorrhage	33(17.6%)	8(13.1%)	25(19.7%)	0.268
Infarction	2(1.1%)	0(0.0%)	2(1.6%)	0.324
Epilepsy	14(7.4%)	6(9.8%)	8(6.3%)	0.387
SMG grading				0.184
I	25(13.3%)	9(14.8%)	16(12.6%)	
II	45(23.9%)	9(14.8%)	36(28.3%)	
III	69(36.7%)	22(36.1%)	47(37.0%)	
IV	35(18.6%)	14(23.0%)	21(16.5%)	
V	14(7.4%)	7(11.5%)	7(5.5%)	
mRS on admission				0.460
0–2	132(70.2%)	45(73.8%)	87(68.5%)	
≥3	56(29.8%)	16(26.2%)	40(31.5%)	
Superficial location	89(47.3%)	27(44.3%)	62(48.8%)	0.558
Eloquence	88(46.8%)	36(59.0%)	64(50.4%)	0.267
Diffuse nidus	41(21.8%)	11(18.0%)	30(23.6%)	0.385
Nidus size ≥3, cm	123(65.4%)	42(68.9%)	82(64.6%)	0.562
Mean size, cm	4.374(5.525)	4.893(5.311)	4.126(2.194)	0.163
No. of pedicles ≥3	141(75%)	46(75.4%)	95(74.8%)	0.995
Pedicle diameter, mostly >1 mm	165(87.8%)	56(91.8%)	109(85.8%)	0.242
Perforating artery supply	54(28.7%)	14(23.0%)	40(31.5%)	0.225
Pedicle from A to P circulation	64(34.0%)	18(29.5%)	46(36.2%)	0.363
No. of draining veins ≥3	75(39.9%)	26(42.6%)	49(38.6%)	0.596
Superficial venous drainage only	99(52.7%)	29(47.5%)	70(55.1%)	0.330
Draining veins extension	112(59.6%)	41(67.2%)	71(55.9%)	0.139
Mean diameter of draining veins, mm	5.987(3.862)	6.006(3.133)	5.978(4.178)	0.963
Presence of aneurysm	41(21.8%)	9(14.8%)	32(25.2%)	0.105
Presence of fistula	7(3.7%)	2(3.3%)	5(3.9%)	0.823

Bold values indicate statistical significance.

### Angiographic and clinical outcomes

**Figure 3:**
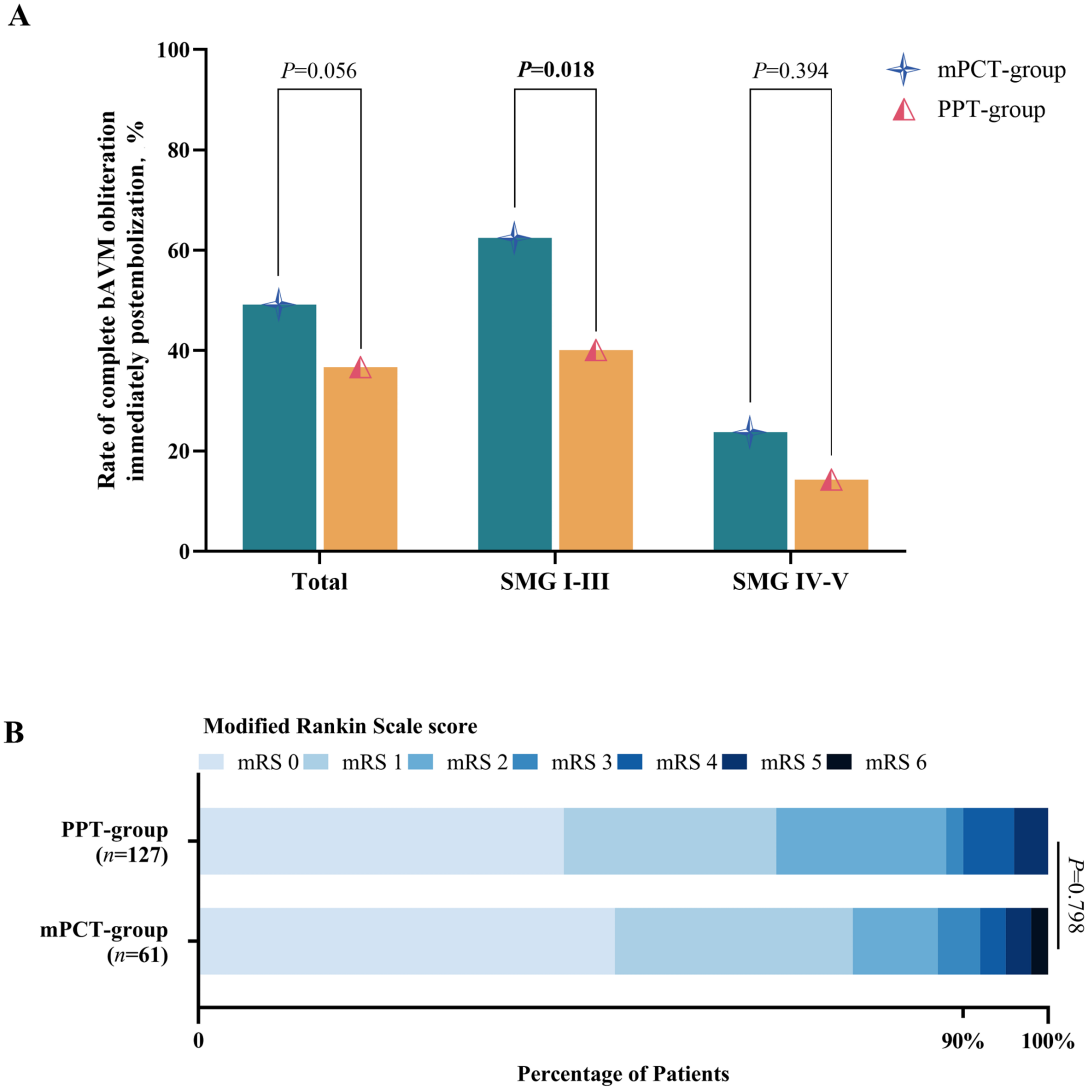
**(A)** Rate of complete obliteration stratified by Spetzler–Martin grade. **(B)** Distribution of 6-month mRS scores in patients treated with mPCT versus PPT.

Immediate post-embolization obliteration rates are summarized in Table 2 and Figure 3A. Complete obliteration was achieved in 49.2% of patients in the mPCT group and 34.6% in the PPT group (*p* = 0.056). Among SMG I-III lesions, the mPCT group had a pivotal higher occlusion rate (62.5% vs. 40.1%; *p* = 0.018). In contrast, for SMG IV-V lesions, the occlusion rate was 9.5% higher in the mPCT group, though not statistically significant (*p* = 0.394). At 3-month follow-up, nidus recurrence was observed in 3.4% of the mPCT group and 4.5% of the PPT group (*p* = 0.795).

**Table 2:** Procedural characteristics and angiographic/clinical outcomes of patients treated with mPCT versus PPT.

Variable	Totals 188	mPCT-group *n* = 61	PPT-group *n* = 127	*P*-value
Angiographic outcomes				0.056
Complete obliteration	74(39.4%)	30(49.2%)	44(34.6%)	
Partial obliteration	114(60.6%)	31(50.8%)	83(65.4%)	
SMG I-III				**0.018**
Complete obliteration	65(46.8%)	25(62.5%)	40(40.1%)	
Partial obliteration	74(53.2%)	15(37.5%)	59(59.6%)	
SMG IV-V				0.394
Complete obliteration	9(18.4%)	5(23.8%)	4(14.3%)	
Partial obliteration	40(81.6%)	16(76.2%)	24(85.7%)	
No. of embolized pedicles				0.286
≤3	61(32.4%)	23(37.7%)	38(29.9%)	
>3	127(67.6%)	38(62.3%)	89(70.1%)	
Complications	42(22.3%)	12(19.7%)	30(23.6%)	0.543
Intraop hemorrhage	10(5.3%)	2(3.3%)	8(6.3%)	0.388
Postop hemorrhage	13(6.9%)	4(6.6%)	9(7.1%)	0.893
Cerebral ischemia	5(2.7%)	2(3.3%)	3(2.4%)	0.715
Transient neurological deficits	10(5.3%)	3(4.9%)	7(5.5%)	0.865
Postop seizures	4(2.1%)	1(1.6%)	3(2.4%)	0.748
Angiographic outcome at 3 months post-op				0.795
Complete obliteration	71(96.0%)	29(96.7%)	42(95.5%)	
Partial obliteration	3(4.0%)	1(3.4%)	2(4.5%)	
mRS score at 6 months post-op				0.798
<3	165(87.8%)	53(86.9%)	112(88.2%)	
≥3	23(12.2%)	8(13.1%)	15(11.8%)	

Bold values indicate statistical significance.

Perioperative complications occurred in 22.3% of patients, a rate comparable to that reported in previous studies. The PPT group experienced more intraoperative (6.3% vs. 3.3%) and postoperative hemorrhage (7.1% vs. 6.6%), while the mPCT group had slightly more cerebral infarctions (3.3% vs. 2.4%). At 6 months, favorable outcomes were similar between groups, with 86.9% in mPCT group and 88.2% in PPT group (Figure 3B).

### Risk factors for incomplete obliteration in the mPCT group

**Figure 4:**
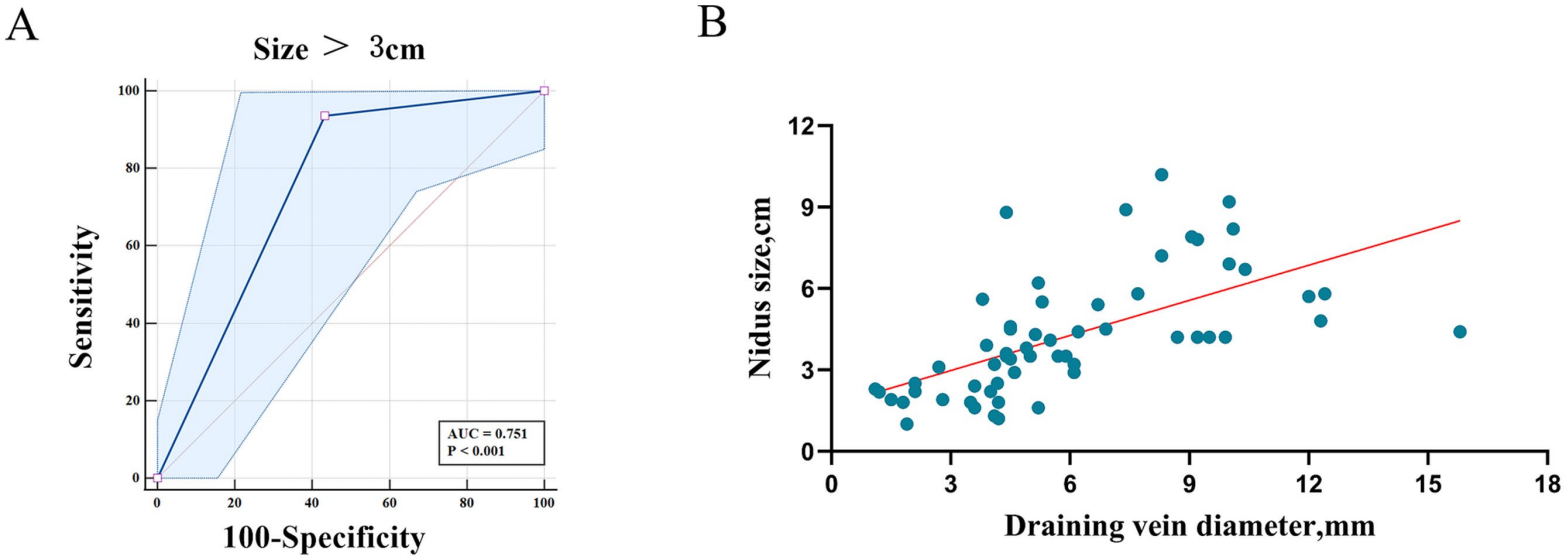
**(A)** ROC curve of nidus size > 3 cm for predicting incomplete obliteration. **(B)** Correlation between draining vein diameter and nidus size, assessed using Spearman’s correlation analysis.

Univariate analysis identified four variables with *p* < 0.10 (age > 18 years, size > 3 cm, number of pedicles ≥ 3, and draining veins extension), which were included in a multivariate logistic regression analysis to explore independent predictors of immediate incomplete obliteration in the mPCT group (Table 3). Multivariate analysis identified nidus size > 3 cm as a significant predictor of incomplete occlusion, with an AUC of 0.751 (Figure 4A). Meanwhile, a detailed analysis of the relationship between draining vein dilatation and the incomplete obliteration at first embolization showed a positive correlation between the draining vein diameter and nidus size. Larger draining veins were associated with larger nidus dimensions, which in turn increased the technical difficulty of achieving complete occlusion during the initial embolization (Figure 4B).

**Table 3:** Risk factors for incomplete obliteration in univariable and multivariable models.

Variable	Incomplete obliteration *n* = 31	Complete obliteration *n* = 30	Univariable		Multivariable	
			*P*-value	Odds ratio (95% CI)	*P*-value	Odds ratio (95% CI)
Male	16(51.6%)	18(60.0%)	0.510	0.711(0.258–1.962)		
Age > 18 yrs	23(74.2%)	29(93.3%)	**0.059**	**0.205(0.040–1.064)**	0.091	0.166(0.021–1.331)
Hemorrhage admission	13(41.9%)	13(43.3%)	0.912	0.944(0.342–2.606)		
History of hemorrhage	5(16.1%)	3(10.0%)	0.482	1.731(0.375–7.988)		
Deep location	19(61.3%)	15(50.0%)	0.376	1.583(0.573–4.378)		
Eloquence	18(58.1%)	18(60.1%)	0.878	0.923(0.333–2.562)		
Compact nidus	24(77.4%)	26(86.7%)	0.352	0.527(0.137–2.031)		
Size >3 cm	29(93.5%)	13(43.3%)	**<0.001**	**18.962(3.811–94.348)**	**0.006**	**14.042(2.126–92.739)**
No. of pedicles ≥3	27(87.1%)	19(63.3%)	**0.038**	**3.908(1.080–14.141)**	0.237	2.635(0.529–13.118)
Pedicle diameter >1 mm	29(93.5%)	27(90.0%)	0.616	1.611(0.250–10.395)		
Perforating artery supply	8(25.8%)	6(20%)	0.591	1.391(0.418–4.634)		
Pedicle from A to P circulation	12(38.7%)	6(20.0%)	0.114	2.526(0.800–7.979)		
No. of draining veins <3	15(48.4%)	20(66.7%)	0.152	0.469(0.166–1.320)		
Deep venous drainage	19(61.3%)	13(43.3%)	0.163	2.071(0.745–5.751)		
Draining veins extension	25(80.6%)	16(53.3%)	**0.027**	**3.646(1.162–11.444)**	0.492	1.783(0.342–9.284)
Presence of aneurysm	5(16.1%)	4(13.3%)	0.759	1.250(0.301–5.186)		
Presence of fistula	1(3.2%)	1(3.3%)	0.981	0.967(0.058–16.192)		

Bold values indicate statistical significance.

## Discussion

The bAVMs are dynamic vascular malformations, often presenting with hemorrhage, seizures, or neurological deficits (2, 24). Managing bAVMs requires a delicate balance between preventing future hemorrhages and minimizing intervention risks (1). Complete obliteration is the only effective way to prevent hemorrhage, as partial occlusion may increase rupture risk due to elevated pressure in the residual nidus (23, 25). Previous reports have demonstrated complete obliteration rates of approximately 50% with Onyx alone, and cure rates exceeding 90% in carefully selected SMG I–II patients (10, 26–28).

However, Onyx also poses challenges, mainly due to reflux that can cause ischemic complications by occluding normal brain-supplying vessels (29, 30). Microcatheter entrapment during Onyx injection remains a concern, as forced removal may lead to vessel rupture and hemorrhage (11). To mitigate these risks, PCT was developed to create a proximal anti-reflux plug to stabilize injections and direct embolic flow into the nidus (15). The original PCT, which used NBCA for plug reinforcement, was limited by a higher risk of catheter adhesion and greater procedural complexity (17, 19). The present study evaluated a further modification, the mPCT, in which a detachable coil plug is reinforced with Onyx rather than NBCA. This modification is intended to enhance plug stability and injection control, while avoiding the adhesion-related risks of cyanoacrylate. Using Onyx instead of NBCA reduces the risk of microcatheter entrapment, compared with coils alone, yields a denser plug that enhances dispersion efficiency. Although coil–Onyx plugs have been applied in practice, evidence from large cohorts remains limited, underscoring the need for further research.

In this series, mPCT achieved higher immediate obliteration rates compared with PPT, particularly for SMG I–III lesions, while complication rates were similar between groups. These findings suggest that mPCT may offer procedural advantages in selected patients, though the overall comparison between groups did not reach statistical significance. Importantly, in higher-grade bAVMs (SMG IV–V), the benefit of mPCT was less apparent, likely reflecting the anatomical complexity, larger nidus volume, and higher hemodynamic stress of these lesions, where safety concerns dictate a more conservative approach.

Compared with balloon-assisted embolization, mPCT provides a stable proximal plug without temporary balloon inflation. In one trial of 20 patients (16 bAVMs, 34 Scepter Mini catheters), the mean feeder diameter was 1.9 ± 0.5 mm, with 60.9% complete obliteration. Four technical issues were attributed to balloon instability, but no balloon-related complications occurred; three patients (15%) had non-procedural complications (31). A multicenter trial of balloon-assisted embolization reported 23 procedures in 19 bAVM patients, achieving 84.6% complete obliteration, though ischemic stroke occurred in 16% and delayed hemorrhage in 10.5%, without permanent deficits (32). Other PCT modifications have also been explored. The sPCT using coils alone was validated for both efficacy and safety in five reported cases, with an average of three nylon coils used per procedure and no reflux beyond the coil plug or adhesion-related issues observed (17). A liquid plug technique with 33% Glubran 2 was applied in 27 cases, achieving good occlusion but with reflux in 4 patients, representing an mPCT variant that forgoes coils and relies solely on liquid embolic (19). The coil-augmented Onyx injection technique (CAIT), reported in 22 bAVM patients, also relies solely on coils to construct the proximal plug without additional reinforcement using NBCA. In this series, CAIT achieved a mean nidus reduction of 96.7% and complete obliteration in 81.8% of cases, with an average Onyx volume of 4.2 mL per procedure (33).

Although these reported occlusion rates appear somewhat higher than those observed in our cohort, it is important to note that they likely reflect more selective case inclusion. In lower-grade lesions (SMG I–II), our immediate complete obliteration rate reached 77.8%, which is comparable to those series. Moreover, the mean Onyx volume used in our mPCT group was 8.88 mL, and the average feeder artery diameter was 2.23 ± 1.01 mm, both indicating that our cases generally involved larger and more complex nidi. The focus of our work, therefore, was to explore the feasibility of mPCT in such challenging lesions, where achieving curative embolization remains most difficult.

Our study also explored factors associated with incomplete obliteration. Multivariable analysis identified nidus size >3 cm as the strongest predictor of incomplete cure with mPCT, consistent with prior studies. Draining vein dilatation showed borderline association, likely reflecting its correlation with nidus size. These findings emphasize that lesion morphology, rather than demographic factors, primarily determines the technical curability of bAVMs. From a mechanistic standpoint, the tighter plug and higher pressure gradient achieved in mPCT may facilitate deeper and more homogeneous Onyx penetration, theoretically lowering the risk of occult residual channels that predispose to recurrence. Although our angiographic follow-up was short, these technical features support the hypothesis of improved long-term durability, which must be tested in longitudinal studies.

To minimize the risk of hemorrhage from premature venous compromise or partial occlusion due to insufficient nidal penetration, we followed Chapot’s principle of progressive embolization—from the periphery toward the center—treating smaller feeders and critical anastomoses first, then addressing the main arterial feeders and primary draining veins, and finally closing the dominant venous outflow (29, 34). In a large multicenter analysis of 846 cases, Baharvahdat et al. (35) reported a bleeding complication rate of 11%, with perioperative arterial perforation (48%) and nidus rupture (52%) as the leading mechanisms. In our experience, the use of detachable-tip microcatheters provides an additional safeguard against microcatheter entrapment and hemorrhagic complications. This technology permits safe retrieval if the catheter tip becomes embedded during Onyx reflux, thereby lowering the risk of vessel injury (36). Complications in our cohort were in line with published series. While overall rates did not differ between groups, mPCT had slightly more cerebral infarctions, all occurring in patients with large lesions requiring longer procedures and higher embolic volumes. Conversely, hemorrhagic events were somewhat more frequent in the PPT group. Given the small numbers, these trends should be interpreted with caution, but they highlight the need for careful case selection and intraoperative monitoring.

This study has several limitations. The retrospective, single-center design inherently risks selection bias, particularly regarding operator discretion in choosing mPCT versus PPT. Although treatment strategies were guided by consensus criteria—such as feeder diameter, number of pedicles, and safety considerations—operator judgment could not be eliminated. Moreover, while most procedures were performed by the same experienced team, inter-operator differences and variations in microcatheter choice may still have influenced outcomes. The relatively small sample size further limits statistical power, raising the possibility of type II error in some subgroup comparisons. Finally, the short angiographic follow-up precludes definitive conclusions on recurrence, underscoring the need for longer surveillance.

## Conclusion

The mPCT and PPT demonstrated similar overall obliteration rates and comparable safety profiles. Notably, in SMG I–III lesions, mPCT achieved a significantly higher rate of immediate complete obliteration, indicating a potential advantage in selected cases. Larger, prospective multicenter studies with long-term follow-up are needed to validate these results and clarify the role of mPCT within the broader spectrum of curative embolization strategies.

## Data Availability

The original contributions presented in the study are included in the article/supplementary material, further inquiries can be directed to the corresponding authors.
